# Endoplasmic Reticulum Stress–Related Genes in Yellow Catfish *Pelteobagrus fulvidraco*: Molecular Characterization, Tissue Expression, and Expression Responses to Dietary Copper Deficiency and Excess

**DOI:** 10.1534/g3.115.019950

**Published:** 2015-08-13

**Authors:** Yu-Feng Song, Zhi Luo, Chao Huang, Qi-Liang Chen, Ya–Xiong Pan, Yi-Huan Xu

**Affiliations:** * Key Laboratory of Freshwater Animal Breeding, Ministry of Agriculture, Fishery College, Huazhong Agricultural University, Wuhan 430070, China; † Freshwater Aquaculture Collaborative Innovative Center of Hubei Province, Wuhan 430070, China

**Keywords:** *Pelteobagrus fulvidraco*, molecular characterization, dietary Cu, ER stress, unfolded protein response

## Abstract

Two endoplasmic reticulum (ER) molecular chaperones [glucose-regulated protein 78 (*grp78*) and calreticulin (*crt*)] and three ER stress sensors [PKR-like ER kinase (*perk*), inositol requiring enzyme (*ire*)-1α, and activating transcription factor (*atf*)-6α] cDNAs were first characterized from yellow catfish, *Pelteobagrus fulvidraco*. The predicted amino acid sequences for the yellow catfish *grp78*, *crt*, *perk*, *ire*-1α, and *atf*-6α revealed that the proteins contained all of the structural features that were characteristic of the five genes in other species, including the KDEL motif, signal peptide, sensor domain, and effector domain. mRNAs of the five genes mentioned above were expressed in various tissues, but their mRNA levels varied among tissues. Dietary Cu excess, but not Cu deficiency, activated the chaperones (*grp78* and *crt*) and folding sensors in ER, and the UPR signaling pathways (*i.e.*, *perk*–*eif*2α and the *ire*1–*xbp1*) in a tissue-specific manner. For the first time, our study cloned *grp78*, *crt*, *perk*, *ire*-1α, and *atf*-6α genes in yellow catfish and demonstrated their differential expression among tissues. Moreover, the present study also indicated differential regulation of these ER stress–related genes by dietary Cu deficiency and excess, which will be beneficial for us to evaluate effects of dietary Cu levels in fish at the molecular level, based on the upstream pathway of lipid metabolism (the ER) and thus provide novel insights regarding the nutrition of Cu in fish.

The endoplasmic reticulum (ER) is an important intracellular organelle responsible for protein and lipid synthesis, xenobiotic detoxification, and cellular calcium storage (Borgese *et al.* 2006; Gorlach *et al.* 2006). Conditions disrupting the ER homeostasis cause accumulation of unfolded and misfolded proteins in the ER lumen, create a state defined as ER stress, and activate a complex signaling network termed unfolded protein response (UPR) (Schröder and Kaufman 2005). UPR is known to trigger a unique signaling cascade from the ER to the nucleus, being characterized by enhanced expression of ER-resident chaperones, such as glucose-regulated protein 78 (*Grp78*) (Kozutsumi *et al.* 1988), and calreticulin (*Crt*) (Caspersen *et al.* 2000). In addition, UPR is characterized by downregulation of protein translation through three ER-transmembrane transducers: PKR-like ER kinase (*Perk*), inositol requiring enzyme (*ire*)-1α, and activating transcription factor (*Atf*)-6α (Harding *et al.* 1999). In the unstressed state, the ER lumenal domains of all three stress sensors are bound by the ER chaperone *grp78*. Upon ER stress, *Grp78* binding to unfolded proteins causes dissociation from three ER-transmembrane transducers, leading to their activation (Bertolotti *et al.* 2000; Oikawa *et al.* 2009). Following activation, UPR signaling pathways act to induce expression of genes that encode functions to ameliorate the stressed state of the ER.

During the past few years, ER stress and UPR-related proteins have been extensively analyzed at the molecular level in mammals. Completely characterized cDNA and genomic sequences of *Grp78*, *crt*, *Perk*, *Ire*-1α, and *Atf*-6α have been previously reported in human, mouse, and rat species (Schröder and Kaufman 2005). However, in fish, to our knowledge, full-length cDNA sequences of the ER stress–related genes (*grp78*, *crt*, *perk*, *ire*-1α, and *atf*-6α) were only determined in very limited fish species, such as grass carp *Ctenopharyngodon idella* (*grp78*) (Zhu *et al.* 2013), rainbow trout *Oncorhynchus mykiss* (*crt*) (Kales *et al.* 2004), Asian seabass *Lates calcarifer* (*crt*) (Bai *et al.* 2012), and medaka *Oryzias latipes* (*atf*6) (Ishikawa *et al.* 2011), although some partial sequences have been reported for other fish species (Ojima *et al.* 2005; Komoike and Matsuoka 2013; Chen *et al.* 2014; Das *et al.* 2015; Li and Li 2015). Although their expression pattern was often covered in these works, study involved in their physiological function was often absent. Thus, identification of ER stress–related genes in other teleosts was a key step for characterizing the role and mechanism of ER stress.

Copper (Cu) is an essential micronutrient for vertebrate animals including fish (Watanabe *et al.* 1997). It has numerous functions in cellular biochemistry including vital roles in cellular respiration, and as a cofactor for more than 30 different enzymes (Watanabe *et al.* 1997). At present, optimal dietary Cu requirement has been determined in many fish species, ranging from 3 to 10 mg Cu kg^−1^ feed, which depends on the species, feeding regime, and life stage (National Research Council 2011). Studies have also shown that overloading of dietary Cu in fish caused toxic syndrome (Berntssen *et al.* 1999; Lundebye *et al.* 1999; Shiau and Ning 2003). In contrast, dietary Cu deficiency has been shown to reduce appetite and growth and cause anemia in several fish (Gatlin and Wilson 1986; Shiau and Ning 2003; Lin *et al.* 2008; Tan *et al.* 2011). Recently, our own studies have pointed out that dietary Cu deficiency and excess could result in the changes of lipid deposition and metabolism in fish (Chen *et al.* 2015). However, the molecular mechanisms of the upstream pathway of lipid metabolism underlying dietary Cu-induced alteration in lipid metabolism have not been elucidated. Studies have pointed out that ER stress is one of the cellular stresses reported to induce lipid accumulation (Lee *et al.* 2008), and ER stress played crucial roles in hepatic lipid metabolism in mammals (Sriburi *et al.* 2004; Rutkowski *et al.* 2008; Kammoun *et al.* 2009). Thus, considering the important role ER plays in lipid metabolism, we hypothesize that ER stress–dependent alteration in lipid homeostasis was the mechanism that underlies the change of lipid deposition of yellow catfish in responses to dietary copper levels.

Yellow catfish *Pelteobagrus fulvidraco*, an omnivorous freshwater fish, is widely distributed in the inland freshwater rivers in China. The fish is regarded as a potential model for studying lipid metabolism in fish because it has relatively high lipid accumulation under natural conditions (Zheng *et al.* 2013a). The cDNA sequences and molecular characterization of many genes have been elucidated in the fish species in our laboratory (Gong *et al.* 2013; Zheng *et al.* 2013a,b; Chen *et al.* 2014; Song *et al.* 2014). Recent studies in our laboratory indicated that dietary Cu deficiency and excess could influence lipid deposition and metabolism in *P. fulvidraco* (Chen *et al.* 2015). However, these studies only determined the change of lipid metabolism–related genes expression and enzyme activities after dietary Cu treatment. The molecular and regulatory mechanisms in the upstream pathway of lipid metabolism under dietary Cu treatment have not been explored. Clearly, an understanding of the molecular basis of ER stress could underpin efforts to address this problem. Therefore, in this study, the full-length cDNA sequences of two ER molecular chaperones (*grp78* and *crt*) and three ER stress sensors (*perk*, *ire*-1α, and *atf*-6α) genes were first cloned and their tissue-specific expressions were determined. Meanwhile, the effect of dietary Cu deficiency and excess on their mRNA levels was explored. The results will be beneficial for us to elucidate the molecular basis of ER stress, and to further evaluate effects of dietary Cu levels in fish at a molecular level, based on the upstream pathway of lipid metabolism, which will greatly extend our understanding of the nutrition of Cu in fish.

## Materials and Methods

Here, two experiments were conducted. The first experiment was involved in the cloning and tissue expression profile analysis of *grp78*, *crt*, *perk*, *ire*-1α, and *atf*-6α transcripts. The second experiment was designed to evaluate a possible transcriptional regulation of *grp78*, *crt*, *perk*, *eif*2α, *ire*-1α, *xbp-1*, and *atf*-6α by dietary Cu deficiency and excess. We ensured that the experiments performed on animals followed the ethical guidelines of Huazhong Agricultural University and Animal Care and Use Committee (IACUC) in Huazhong Agricultural University specifically approved this study.

### Experiment 1: *grp78*, *crt*, *perk*, *ire*-1α, and *atf*-6α, cDNAs cloning and mRNA expression patterns of various tissues

#### Experimental animals:

Yellow catfish (1.83 ± 0.05 g, mean ± SEM) for cloning the *grp78*, *crt*, *perk*, *ire*-1α, and *atf*-6α genes were obtained from a local fish pond (Wuhan, China) and transferred to indoor fiberglass tanks (400 liters in water volume) for 2-wk acclimation. During the acclimation period, the fish were fed to apparent satiation twice daily with a commercial pellet diet. The experiment was conducted at ambient temperature and subjected to natural photoperiod (approximately 14 hr light/10 hr darkness). Water quality parameters were monitored twice per week in the morning. The following parameters were used: water temperature, 27.9 ± 1.2°; pH, 7.66 ± 0.11; dissolved oxygen, 6.34 ± 0.15 mg liter^−1^; and total hardness, 151.8 ± 1.1 mg liter^−1^ as CaCO_3_. Fish were anesthetized with 3-aminobenzoic acid ethyl ester methanesulfonate (MS-222 at 10 mg liter^−1^) and killed by decapitation. Skeletal muscle, gill, head kidney, mid-intestine, brain, spleen, liver, heart, testis, and ovary from six fish were dissected and immediately prepared for RNA isolation (n = 6).

#### RNA isolation and first-strand cDNA synthesis:

Each sample was pulverized with its own mortar and pestle, and the mortars and pestles were heated at 230° for 8 hr to inactivate RNases. Total RNA was extracted using TRIzol RNA reagent (Invitrogen, USA) based on the acid guanidinium thiocyanate-phenol-chloroform extraction method, according to the manufacturer’s instructions. The quality of total RNA was checked by agarose gel electrophoresis. The concentration and the ratio (between 1.8 and 2.0) of OD_260_/OD_280_ of total RNA were determined using a Nanodrop ND-2000 spectrophotometer (Thermo Fisher Scientific, USA). Total RNA (1 μg) was reverse-transcribed to cDNA with oligo-dT primers and a cDNA Synthesis Kit (TaKaRa, Japan) according to manufacturer’s instructions.

#### Cloning and sequencing of grp78, crt, perk, ire-1α, and atf-6α cDNAs:

In the present study, we used the liver of yellow catfish to clone the cDNA sequences of the five genes. Degenerate primers (Table 1) designed based on the most conserved regions of the fish *grp78*, *crt*, *perk*, *ire*-1α, and *atf*-6α sequences available in the GenBank and Ensembl databases were used to amplify partial cDNA fragments of *grp78*, *crt*, *perk*, *ire*-1α, and *atf*-6α. The PCR program consisted of initial denaturing for 4 min at 94° followed by 30 cycles of 94° for 30 sec, 55° for 30 sec, 72° for 1 min, and a final elongation step at 72° for 5 min, with 500 ng cDNA template, 0.2 μM primer, 5 μl 10 × Ex Taq Buffer, 2.5 mM dNTP, and 1.25 units Ex Taq polymerase (TaKaRa, Japan). The target fragments were purified using the EZNA gel extraction kit (Omega, USA) according to manufacturer’s instructions, and then subcloned into the pMD 19-T cloning vector (TaKaRa, Japan). Positive clones containing inserts of an expected size were sequenced with sanger dideoxy mediated chain termination method (Sangon, China).

**Table 1:** Nucleotide sequences of the primers used for the cDNAs cloning of *grp78*, *crt*, *perk*, *ire*-1α, and *atf*-6α genes

Primers	Sequences (5′-3′)
**Primers for partial fragment**	
*grp78*- F	AGAACACVGTYTTYGATGCC
*grp78*-R	TCCMACDGTCTCAATGCC
*crt*-F	RGATGCYCGYTTYTATGC
*crt*-R	GGYTTCCAYTCSCCCTKRTA
*perk*-F	AGTGYTTAGGTSGAGGCVG
*perk*-R	GAGSAGTGARAGAGGMTGG
*ire-1α*-F	ACTCTRACCTCCTYTTTTG
*ire-1α*-R	CCMCTRTCATTYACCTTCT
*atf-6α*-F	TGGTMCTTGYTTTCCTGGC
*atf-6α*-F	TCMTCASCTCAATCTCCTT
**Primers for 3′ RACE PCR**	
3′ GS-*grp78* 01-F	TGAAGACTTTGACCAGCGTG
3′ GS-*grp78* 11-F	CTGGACAGGAGGATACAGGAA
3′ GS-*crt 01*-F	GGATGATGAATACACCCACC
3′ GS-*crt 11*-F	GATGGATGGAGAATGGGAGC
3′ GS-*perk* 01-F	CCAATCAGATGCGGTGTCC
3′ GS-*perk* 11-F	GGACAAGTGGGTACTAAAC
3′ GS-*ire-1α* 01-F	CAAACATCCCTTTTTCTGG
3′ GS-*ire-1α* 11-F	ACATCCCGCTTTCCCCACCT
3′ GS-*atf-6α* 01-F	CTTGAGGAACACCATCCCA
3′ GS-*atf-6α* 11-F	CTGCTTATTCACTGCATCA
3′ RACE Outer	TACCGTCGTTCCACTAGTGATTT
3′ RACE Inner	CGCGGATCCTCCACTAGTGATTTCACTATAGG
**Primers for 5′ RACE PCR**	
5′ GS-*grp78* 01-R	CAGACCATAGGCGATAGCAG
5′ GS-*grp78* 11-R	TTTCCAAGGTAAGCCTCTGC
5′GS-*crt 01*-R	TTACCTTTGTAGGCAGGATTG
5′ GS-*crt 11*-R	CTCCCATTCTCCATCCATCT
5′ GS-*perk 01*-R	CAGGGCTTTGGGTTTAGAT
5′ GS-*perk 11*-R	TGTAGATTCCCAGACCATA
5′ GS-*ire-1α 01*-R	CACCACGGGAGAGTCATAG
5′ GS-*ire-1α 11*-R	TTTCGTCAGTCCCTCATTG
5′ GS-*atf-6α 01*-R	TGAATGGGTCTCATAACAG
5′ GS-*atf-6α 11*-R	AACTCTTCCGCATCTCCCA
5′ RACE Outer	CATGGCTACATGCTGACAGCCTA
5′ RACE Inner	CGCGGATCCACAGCCTACTGATGATCAGTCGATG

Mixed bases: R-A/G; Y-C/T; M-A/C; K-G/T; S-G/C; W-A/T; H-A/T/C; B-G/T/C; V-G/A/C; D-G/A/T.

To obtain the 3′ and 5′ end sequences of *grp78*, *crt*, *perk*, *ire*-1α, and *atf*-6α, nested 3′ and 5′ RACE PCR were performed with a SMART RACE cDNA Amplification Kit (Clontech, USA) using the DNase treated total RNA, with 1 μl 10 × Reaction Buffer, 1 units DNase I for 1 μg RNA incubated at 37° for 15 min, then with 1 μl 25 mM EDTA incubated at 65° for 15 min to inactivate the DNase I. In the first PCR, the cDNA was amplified with two outer primer sets (Table 1) and Universal Primer Mix (provided in the kit) using Advantage 2 PCR Kit (Clontech, USA) with 200 ng cDNA template, 10 μM primer, 4 μl 10 × LA PCR Buffer, 25 mM MgCl_2_, and 1.25 units LA Taq (TaKaRa, Japan). In the second PCR, inner primer sets (Table 1) and Nested Universal Primer (provided in the kit) were used, with 1 μl 1^st^ cDNA product, 2.5 mM dNTP, 10 μM primer, 5 μl 10 × LA PCR Buffer, 25 mM MgCl_2_, and 2.5 units LA Taq (TaKaRa, Japan). The PCR parameters were 30 cycles at 94° for 30 sec, 55° for 30 sec, and 72° for 1 min, with an additional initial 3-min denaturation at 94° and a 10-min final extension at 72°. The RACE products were sequenced as described above.

#### Sequence analysis:

The core fragment, 3′ end and 5′ end sequences were assembled using SeqMan II software in DNASTAR PACKAGE to obtain full-length *grp78*, *crt*, *perk*, *ire*-1α, and *atf*-6α cDNAs. The sequences were edited and analyzed using the program EDITSEQ of DNASTAR package to search for the open reading frame (ORF) and then translated into amino acid sequences using standard genetic codes. The nucleotide sequences were compared with DNA sequences present in the GenBank database using BLAST network service at the NCBI (http://blast.ncbi.nlm.nih.gov/). Sequence alignments and percentage of amino acid conservation were assessed with the Clustal-W multiple alignment algorithm. For phylogenetic analysis, multiple sequence alignments were made with MAFFT (Katoh *et al.* 2002) using an amino acid model at the GUIDANCE web server (http://guidance.tau.ac.il/) (Penn *et al.* 2010), which pruned unreliably aligned regions by rejecting columns with confidence scores below 0.93. The phylogenetic tree was constructed with MEGA 5.0 (Tamura *et al.* 2011) by the neighbor-joining (NJ) method based on the JTT+G model (Jones *et al.* 1992), the best-fit model of sequence evolution obtained by ML model selection. The confidence of each node was assessed by 1000 bootstrap replicates.

### Experiment 2: responses of mRNA expression of *grp78*, *crt*, *perk*, *eif*2α, *ire*-1α, *xbp-1*, and *atf*-6α to dietary Cu deficiency and excess

#### Diet preparation:

Three experimental diets, in agreement with the study by Chen *et al.* (2015), were formulated with CuSO_4_⋅5H_2_O supplemented at levels of 0, 0.013, and 0.39 g kg^−1^ diet at the expense of cellulose (Table 2). Different Cu contents were added to the diets based on our recent study (Tan *et al.* 2011) to produce three different dietary Cu groups (Cu deficiency, adequate Cu, and Cu excess, respectively). The formulation of the experimental diets was detailed in the work by Chen *et al.* (2015). The formulated diets were dried at 80° in an oven until the moisture was reduced to less than 10%. The dry pellets were placed in plastic bags and stored at 20° until fed. Final Cu concentrations in the experimental diets were analyzed in triplicate using graphite furnace atomic absorption spectrometry (GFAAS) (Chen *et al.* 2015), and the contents were 0.76 (Cu deficiency), 4.18 (adequate Cu), and 92.45 (Cu excess) mg Cu kg^−1^ diet, respectively.

**Table 2:** Feed formulation and proximate analysis of experimental diets (Chen *et al.* 2015)

Ingredients (g kg^−1^)	Adequate Cu	Cu Deficiency	Cu Excess
Casein	320	320	320
Gelatin	80	80	80
Fish oil	25	25	25
Corn oil	50	50	50
Wheat flour	250	250	250
Ascorbyl-2-polyphosphate	10	10	10
NaCl	10	10	10
NaH_2_PO_4_⋅2H_2_O	10	10	10
CuSO_4_⋅5H_2_O	0.013	0	0.39
Vitamin premix	5	5	5
Mineral premix (Cu-free)	5	5	5
Betaine	10	10	10
Cellulose	224.987	225	224.61
Proximate analysis (% dry matter basis)			
Moisture	8.63	8.58	8.92
Crude protein	34.38	34.21	34.29
Lipid	7.63	7.59	7.72
Cu (mg kg^−1^)	4.18	0.76	92.45

Vitamin premix according to Tan *et al.* (2010); Mineral premix according to Tan *et al.* (2010) without Cu addition. CuSO_4_·5H_2_O(≥99.0% in purity): Sinopharm Chemical Reagent Co. Ltd., Shanghai, China.

#### Experimental procedures:

Experimental procedures are in agreement with these in our parallel study (Chen *et al.* 2015). Briefly, yellow catfish were obtained from a local fish pond (Wuhan, China) and transferred to indoor fiberglass tanks (400 liters in water volume) for 2-wk acclimation. At the beginning of the experiment, 30 uniform-sized fish (1.83 ± 0.05 g; mean ± SEM) were stocked in each fiberglass tank (the loading of fish was approximately 0.183 g l^−1^) and subjected to natural photoperiod (approximately 14 hr light/10 hr darkness). Each diet was assigned to three tanks in a completely randomized design, with nine tanks for the experiment. Water in each tank was renewed 80% twice daily, before feeding. They were provided with continuous aeration to maintain the dissolved oxygen level near saturation. The fish were fed to apparent satiation twice daily at two equal meals (09:00 and 16:30 hr) during the week. Care was taken to ensure that no noneaten food remained in the tanks during feeding, thus leaching of CuSO_4_ into water was very low and negligible. Cu concentrations in water samples collected 10 min before and after feeding remained low throughout the experiment (1.7 ± 0.2 μg 1^−1^). The experiment continued for 8 wk. At the end of the 8-wk period, 24 hr after the last feeding, all fish were killed with MS-222 (10 mg l^−1^). Three fish per tank were randomly selected. After the skin was rapidly removed from muscle, the liver and skeletal muscle above the lateral line were removed immediately using sterile forceps, frozen in liquid nitrogen, and stored at −80° (not longer than 2 wk) for total RNA extraction (n = 3 replicate tanks, and three fish were sampled for each tank).

#### Quantitative real-time PCR:

Analyses on the distribution and mRNA expression levels of *grp78*, *crt*, *perk*, *eif*2α, *ire*-1α, *xbp-1*, and *atf*-6α were conducted by quantitative real-time PCR (qPCR) method. Primers used for qPCR are shown in Table 3. Total RNA extraction and first strand cDNA synthesis were performed according to previously described methods (Chen *et al.* 2014). The cDNA synthesis reactions were diluted to 200 μl in water. Real-time PCR was performed in a 20 μl reaction mixture including 10 μl SYBR Premix Ex TaqTM II (2×), 0.4 μl each primer (25 μM), 1 μl diluted first-strand cDNA (5 ng) product, and 8.2 μl ddH_2_O. Reactions were based on a three-step method as followed: at 95° for 30 sec, 40 cycles of 5 sec at 95°, 10 sec at 57°, and 30 sec at 72°. All reactions were performed in duplicate and each reaction was verified to contain a single product of the correct size by agarose gel electrophoresis. A nontemplate control and dissociation curve were performed to ensure that only one PCR product was amplified and that stock solutions were not contaminated. A 10-fold dilution series was created from a random pool of cDNA from our sample groups ranging from ×1 dilution to ×100,000 dilutions. The PCR efficiency and correlation coefficients of each primer pair were generated using the slopes of the standard curves. The amplification efficiencies of all genes were approximately equal and ranged from 98 to 103%. Because housekeeping gene sequences were not available in *P. fulvidraco*, a set of eight housekeeping genes (β-*actin*, *gapdh*, *ef1A*, *18s rRNA*, *hprt*, *b2m*, tuba, *and rpl17*) were selected from the literature (Zhao *et al.* 2011) to test their transcription stability. The relative expression levels were calculated using the 2^−ΔΔCt^ method (Livak and Schmittgen 2001) when normalizing to the geometric mean of the best combination of two genes (β-*actin* and *gapdh*, M = 0.27) as suggested by geNorm (Vandesompele *et al.* 2002). Prior to the analysis, experiments were performed to check the stability of housekeeping genes, from which β-*actin* and *gapdh* showed the most stable level of expression across the experimental conditions (Chen *et al.* 2014).

**Table 3:** Primers used for real-time PCR analysis

Genes	GenBank Accession No.	Forward Primer (5′-3′)	Reverse Primer (5′-3′)	Size (bp)
*grp78*	KM114873	GCTCCACTCGTAT	TCCGTAAGCCACA	101
		CCCCAA	GCCTCA	
*crt*	KM114875	CGTGAAGAAGAG	AGAATAGGAGGCA	185
		GACAAGA	TAGCAG	
*perk*	KP687344	GAAAAATAACATG	GCCGAGGCACCAT	197
		GTGCCTCGG	GTTATTTTTC	
*eif*2α	KR231690	AGGATGTGGTGAT	CGATGCGGATAAG	147
		GGTGAA	TTTGTT	
*ire*-1α	KP687345	CCTACTTCACATCC	AGTTCGCTTGACT	168
		CGCTT	TTGCTC	
*xbp-1*	KR231691	GTGCTTCTCATTTC	ACTCTGTTCTTCA	143
		TTCATC	GTTTCC	
*atf*-6	KP687343	CAGTAAGAAGGCG	TGGTGAGGGGCG	102
		GAAGTG	TAGTAGAC	
β-*actin*	EU161066	GCACAGTAAAGGC	ACATCTGCTGGAA	136
		GTTGTGA	GGTGGAC	
*gapdh*	KP938521	CACTGCCACCCAG	AGGGACACGGAA	143
		AAGACA	AGCCAT	

### Statistical analysis

Quantitative data were expressed as means ± SEM. Prior to statistical analysis, all data were tested for normality of distribution using the Kolmogorov–Smirnov test. The homogeneity of variances among the different tissues was tested using the Barlett’s test. Data were then subjected to one-way ANOVA and Tukey’s multiple range tests. Differences between control (adequate Cu) and individual Cu-treated groups (Cu deficiency and Cu excess) were analyzed by student’s *t*-test for independent samples. Difference was considered significant at *P* < 0.05. All statistics were performed using the SPSS 17.0 for Windows (SPSS, Chicago, IL, USA).

### Data availability

File S1 contains detailed data about tissue expression mRNA of *grp78*, *crt*, *perk*, *ire*-1α, and *atf*-6α. File S2 contains detailed about effect of dietary Cu deficiency and excess on epression of *grp78*, *crt*, *perk*, *eif*2α, *ire*-1α, *xbp*-1α, and *atf*-6α.

## Results

### Molecular characterization of *grp78*, *crt*, *perk*, *ire*-1α, and *atf*-6α cDNAs

**Figure 1:**
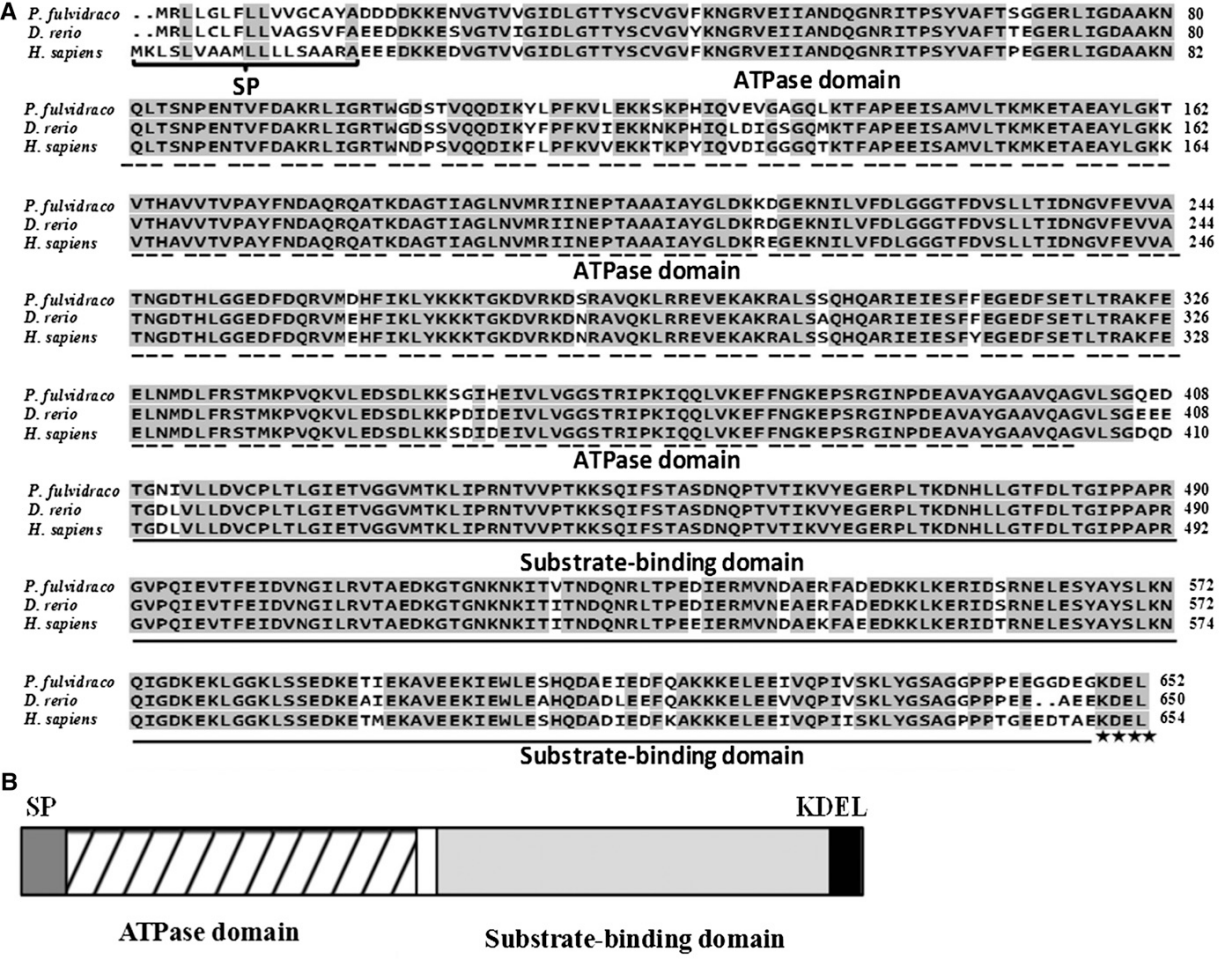
(A) ClustalX alignment of the deduced amino acid sequence of *grp78* from *P. fulvidraco* and other species. The identical residues are shaded dark gray. Characteristic features are denoted as follows: SP (signal peptide) brace; C-terminal KDEL motif, an ER-retrieval sequence, asterisk; ATPase domain, dotted line; substrate-binding domain, solid line. Deduced amino acid sequences were obtained from *D. rerio* (GenBank/EMBL accession no. ENSDART00000010079) and *H. sapiens* (M19645). (B) The predicted peptide features of *grp78*. SP, signal peptide.

**Figure 2:**
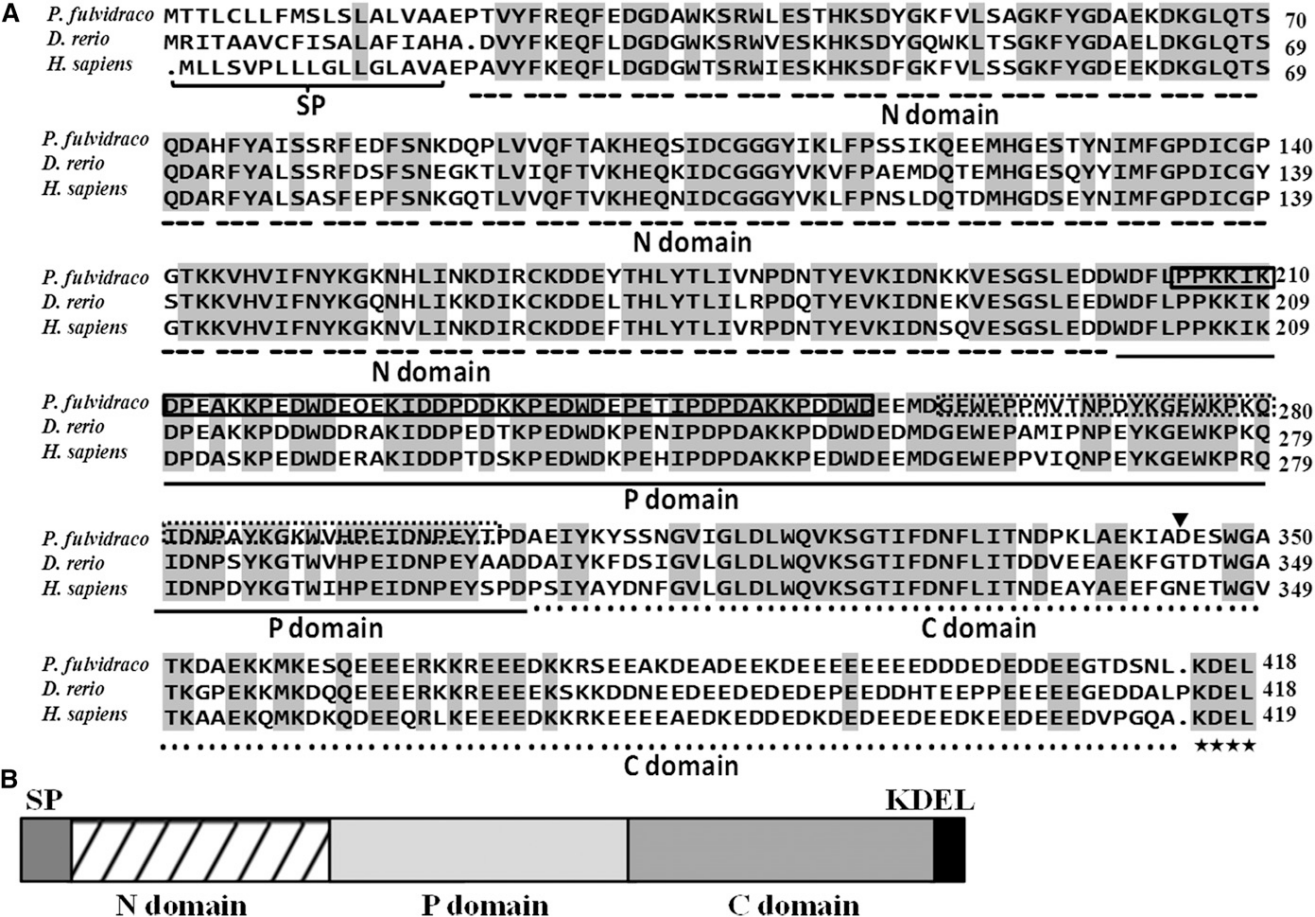
(A) ClustalX alignment of the deduced amino acid sequence of *crt* from *P. fulvidraco* and other species. The identical residues are shaded dark gray. Characteristic features are denoted as follows: SP (signal peptide) brace; *N*-glycosylation site, triangle; C-terminal KDEL motif, an ER-retrieval sequence, asterisk; N domain, dashed line; P domain, solid line; C domain, dotted line; triplicate repeats A, solid lines box; triplicate repeats B, dotted lines box. Deduced amino acid sequences were obtained from *D. rerio* (GenBank accession no. NM_131047) and *H. sapiens* (NM_004343). (B) The predicted peptide features of *crt*. SP, signal peptide.

**Figure 3:**
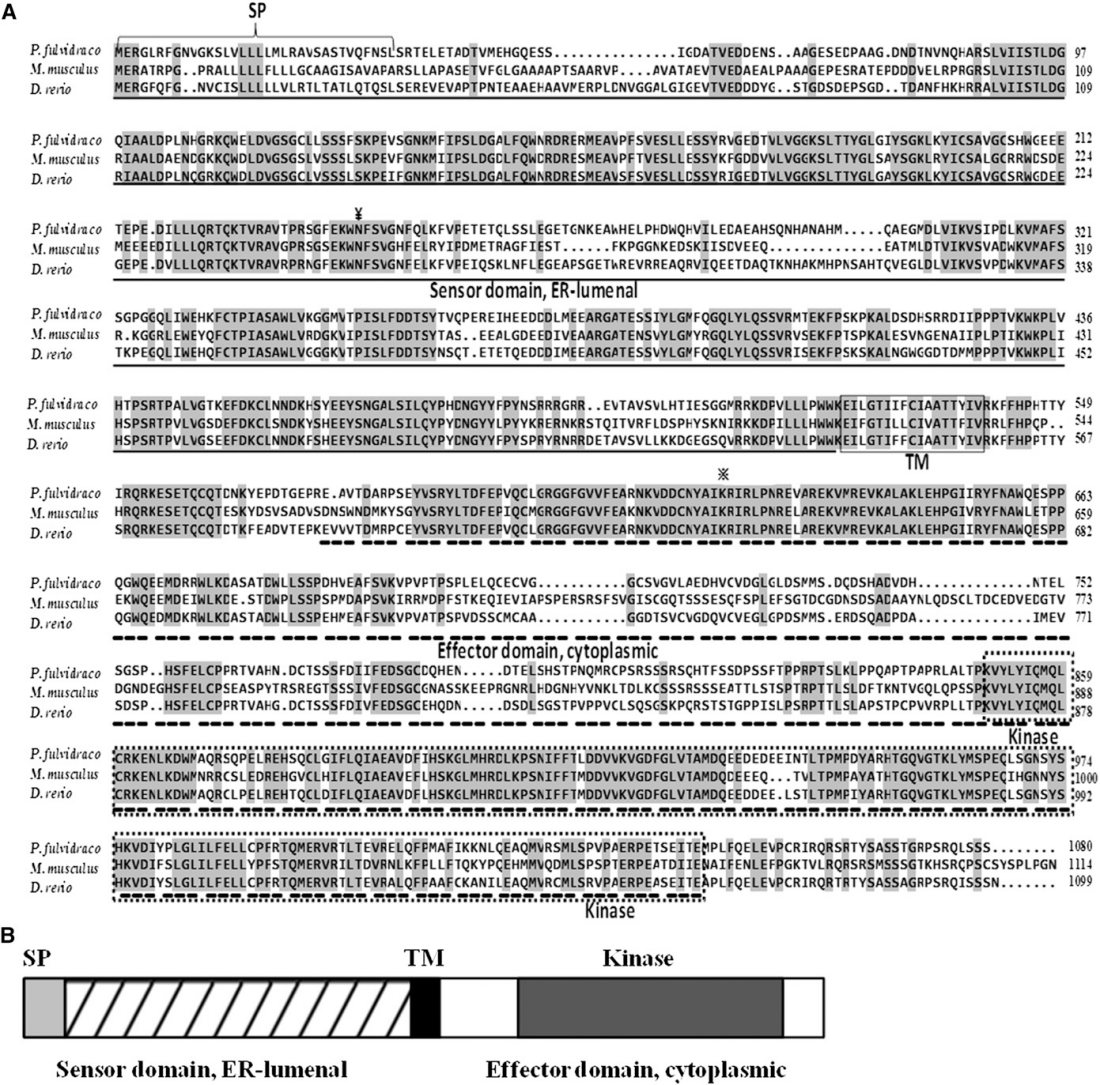
(A) ClustalX alignment of the deduced amino acid sequence of *perk* from *P. fulvidraco* and other species. The identical residues are shaded dark gray. Characteristic features are denoted as follows: SP (signal peptide) brace; *N*-linked glycosylation site, ¥; the invariant lysine, ※; TM (transmembrane domain), solid lines box; Kinase (protein kinase), dotted lines box; stress sensing domain, solid line; effector domain, dotted line. Transmembrane regions were predicted from a hydropathy plot taking peaks with scores above 1.6 using a scan window size of 18 (Kyte and Doolittle 1982). Deduced amino acid sequences were obtained from *M. musculus* (GenBank accession no. NM_010121) and *D. rerio* (XM_005156585). (B) The predicted peptide features of *perk*. SP, signal peptide; TM, transmembrane domain; Kinase, kinase protein.

**Figure 4:**
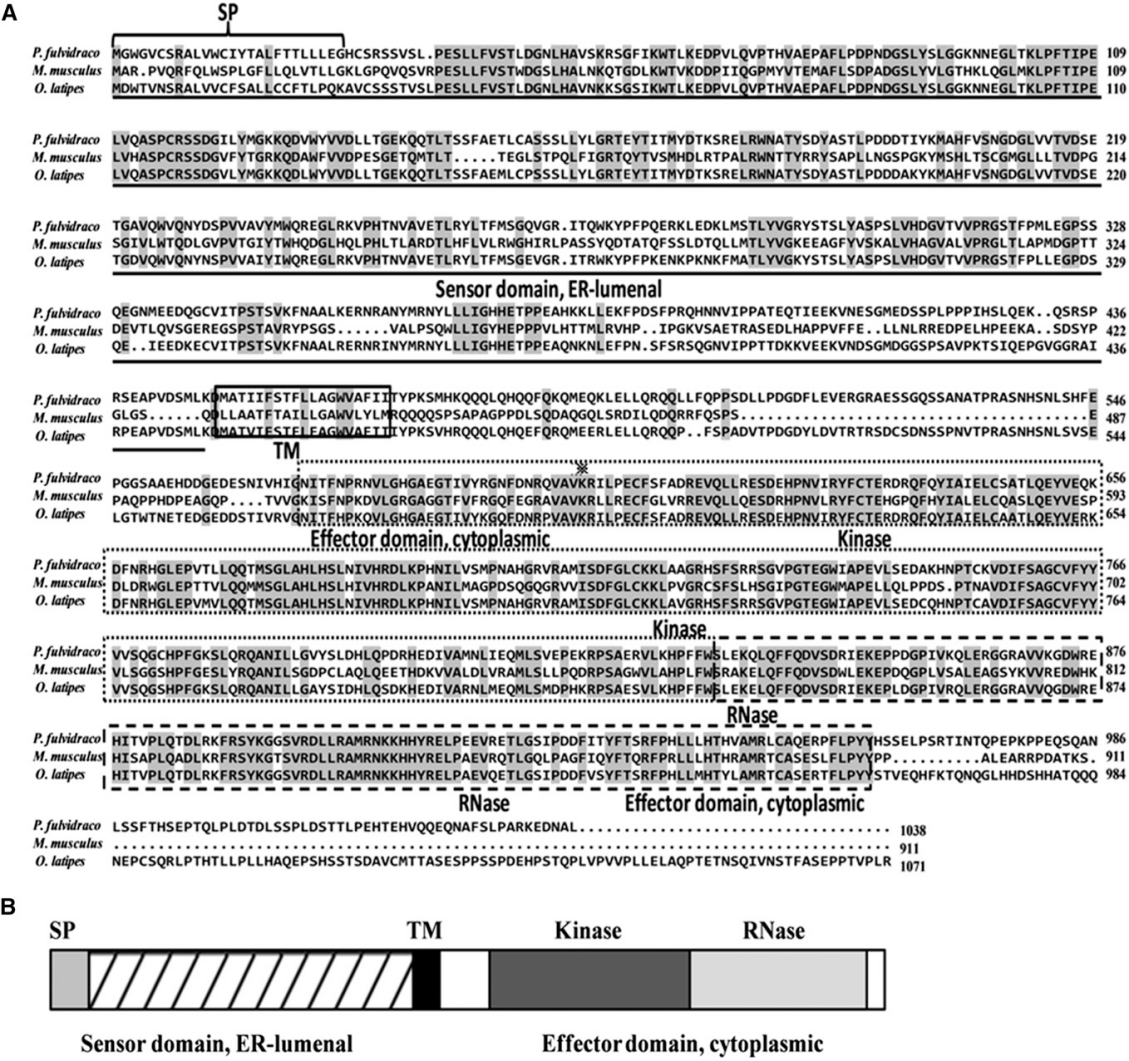
(A) ClustalX alignment of the deduced amino acid sequence of *ire*-1α from *P. fulvidraco* and other species. The identical residues are shaded dark gray. Characteristic features are denoted as follows: SP (signal peptide) brace; TM (transmembrane domain), solid lines box; Kinase (protein kinase), dotted lines box; RNase (ribonuclease), bold dotted lines box; the conserved lysine in kinase domain II, ※; stress sensing domain, solid line; effector domain, dotted line. Transmembrane regions were predicted from a hydropathy plot taking peaks with scores above 1.6 using a scan window size of 18 (Kyte and Doolittle 1982). Deduced amino acid sequences were obtained from *M. musculus* (GenBank accession no. AF071777) and *O. latipes* (AB667982). (B) The predicted peptide features of *ire*-1α. SP, signal peptide; TM, transmembrane domain; Kinase, protein kinase; RNase, ribonuclease.

**Figure 5:**
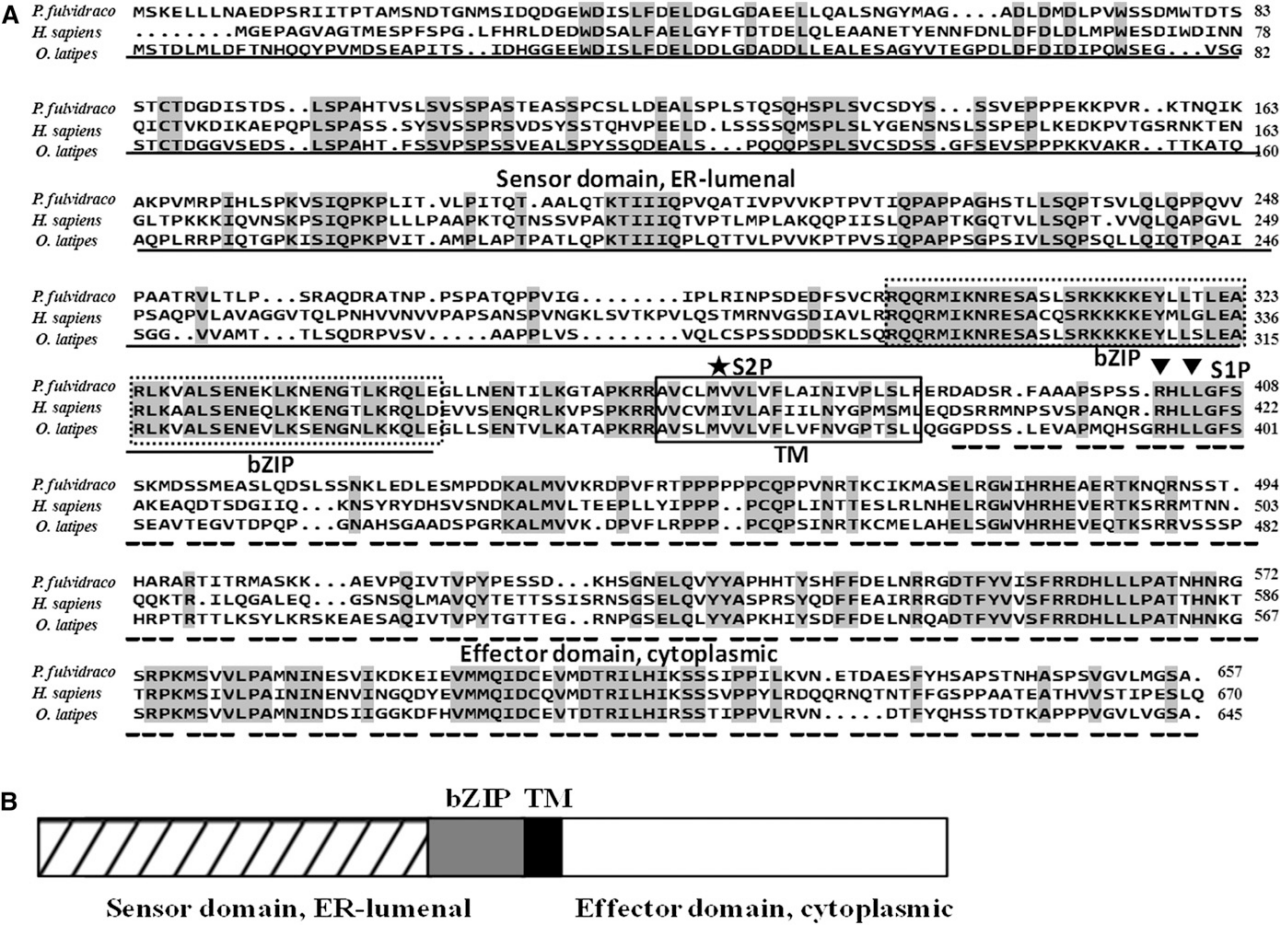
(A) ClustalX alignment of the deduced amino acid sequence of *atf*-6α from *P. fulvidraco* and other species. The identical residues are shaded dark gray. Characteristic features are denoted as follows: bZIP (basic leucine zipper), dotted lines box; TM (transmembrane domain), solid lines box; S1P, (Site-1 cleavage site) triangle; S2P, (Site-2 cleavage site), asterisk; stress sensing domain, solid line; effector domain, dotted line. Transmembrane regions were predicted from a hydropathy plot taking peaks with scores above 1.6 using a scan window size of 18 (Kyte and Doolittle 1982). Deduced amino acid sequences were obtained from *H. sapiens* (GenBank accession no. AB015856) and *O. latipes* (NM_001278901). (B) The predicted peptide features of *atf*-6α. bZIP, basic leucine zipper; TM, transmembrane domain.

In the present study, by RT-PCR, 3′- and 5′-RACE PCR, we obtained the full-length cDNA sequences of *grp78*, *crt*, *perk*, *ire*-1α, and *atf*-6α genes, which were shown to be 2544 bp, 1678 bp, 3776 bp, 3440 bp, and 2785 bp, respectively (Table 4). Sequencing revealed that their cDNAs included an ORF of 1956 bp, 1254 bp, 3240 bp, 3113 bp, and 1971 bp, respectively, encoding a protein of 652 amino acids, 418 amino acids, 1080 amino acids, 1038 amino acids, and 657 amino acids, respectively. The protein sequence of *P. fulvidraco grp78* possessed all the characteristic features of *grp78*, including signal peptide, C-terminal KDEL motif, ATPase domain, and substrate-binding domain (Figure 1). The protein sequence of yellow catfish *crt* possessed all the characteristic features of *crt*, including signal peptide, *N*-glycosylation site, and C-terminal KDEL motif (Figure 2). The predicted amino acid sequence for the yellow catfish *perk*, *ire*-1α, and *atf*-6α possessed all the characteristic features of ER stress–sensing genes, including signal peptide, transmembrane domain, protein kinase, ribonuclease, sensor domain, and effector domain (Figure 3, Figure 4, and Figure 5).

**Table 4:** Information for *grp78*, *crt*, *perk*, *ire*-1α, and *atf*-6α cDNAs cloned from yellow catfish

	*grp78*	*crt*	*perk*	*ire*-1α	*atf*-6α
Length (bp)	2544	1678	3776	3440	2785
5′- UTR (bp)	143	126	181	157	106
3′- UTR (bp)	445	298	355	169	708
ORF (bp)	1956	1254	3240	3113	1971
No. of amino acids	652	418	1080	1038	657

Alignment of predicted polypeptide sequences showed that the amino acid sequences of yellow catfish *grp78*, *crt*, *perk*, *ire*-1α, and *atf*-6α were similar to those from other fish and mammals, exhibiting 91.9–94.9%, 69.9–84.8%, 56.8–74.4%, 65.1–81.7%, and 40.2–65.0% amino acid sequence identities, respectively (Table 5).

**Table 5:** Amino acid sequence identity of *grp78*, *crt*, *perk*, *ire*-1α, and *atf*-6α between yellow catfish and other species (%).

	Zebrafish	Medaka	*Xenopus*	Turkey	Horse	Mouse	Human
*grp78*	93.2	92.6	91.1	94.9	91.2	91.1	91.3
*crt*	70.0	69.9	73.3	84.8	73.8	73.8	73.4
*perk*	74.4	68.6	56.8	61.9	61.5	57.9	58.6
*ire*-1α	81.1	81.7	65.1	72.3	74.4	72.0	73.6
*atf*-6α	65.0	57.5	40.4	44.2	43.8	44.1	43.8

EMBL databases accession numbers: *grp78* (ENSDARG00000004665, ENSORLG00000006886, ENSXETG00000016838, ENSMGAG00000008510, ENSECAG00000024205, ENSMUSG00000026864, ENSG00000044574); *crt* (ENSDARG00000043276, ENSORLG00000002923, ENSXETG00000007937, ENSMGAG00000002470, ENSECAG00000008164, ENSMUSG00000003814, ENSG00000179218); *perk* (ENSDARG00000062139, ENSORLG00000017993, ENSXETG00000001926, ENSMGAG00000013189, ENSECAG00000020856, ENSMUSG00000031668, ENSG00000172071); *ire*-1α (ENSDARG00000013997, ENSORLG00000008940, ENSXETG00000030398, ENSMGAG00000007831, ENSECAG00000011262, ENSMUSG00000020715, ENSG00000178607); *atf*-6α (ENSDARG00000012656, ENSORLG00000016676, ENSXETG00000004747, ENSMGAG00000005185, ENSECAG00000022307, ENSMUSG00000026663, and ENSG00000118217). The order of accession numbers of each gene corresponds to zebrafish, medaka, xenopus, turkey, horse, mouse, and human, respectively.

**Figure 6:**
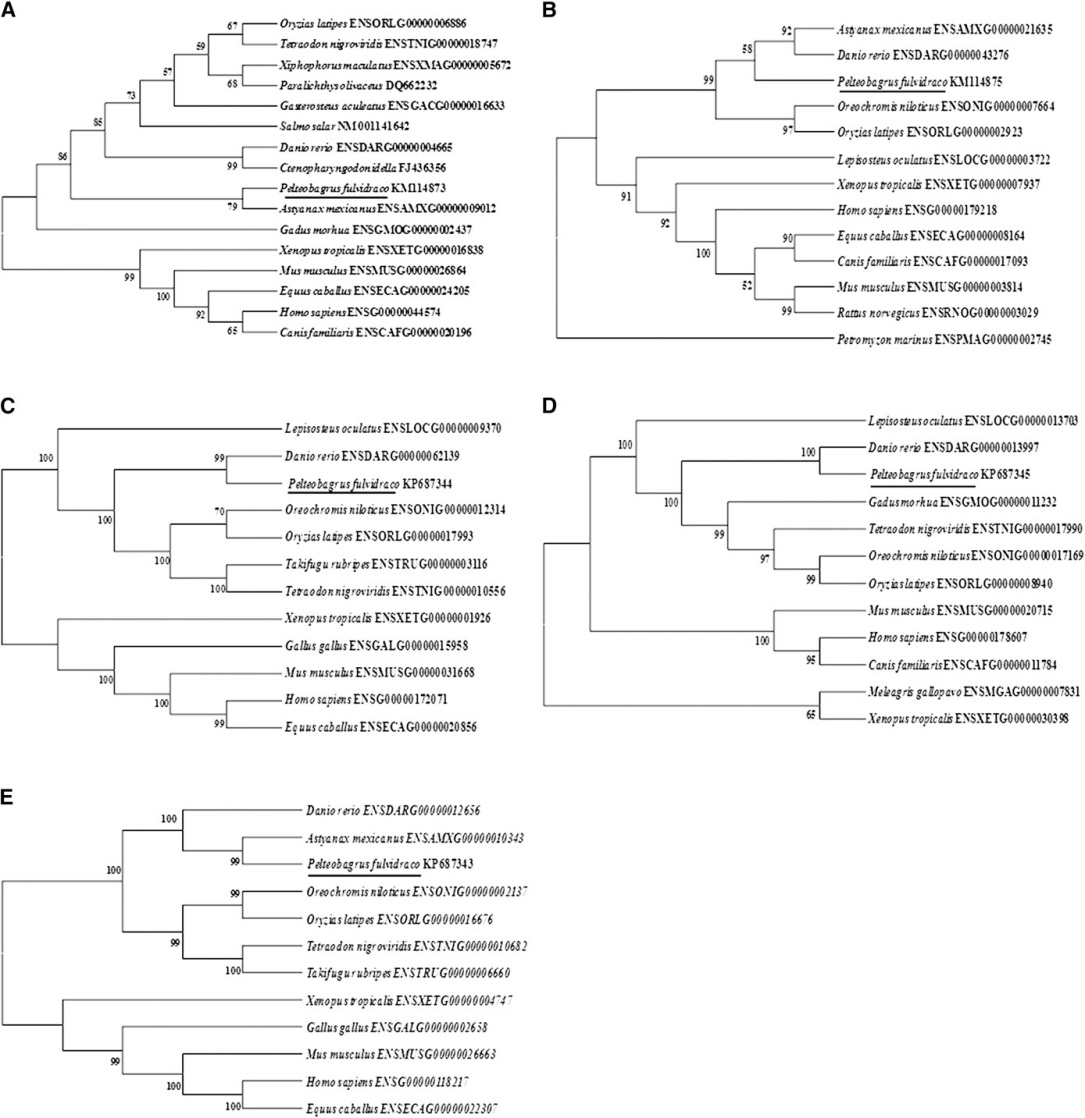
Phylogenetic trees based on the amino acid sequences of *grp78* (A), *crt* (B), *perk* (C), *ire*-1α (D), and *atf*-6α (E) from yellow catfish and the other vertebrate species constructed by neighbor-joining method in MEGA 5.0 (Tamura *et al.* 2011) based on the JTT+G model (Jones *et al.* 1992) with 1000 bootstrap replicates. Accession numbers are shown next to each species. Numbers above branches indicate bootstrap support percentage over 50% in 1000 replicates.

The phylogenetic analysis showed that all teleost *grp78* formed an independent cluster, whereas amphibian and mammalian *grp78* formed another cluster. The *P. fulvidraco grp78* was closer with *Astyanax mexicanus* and more distant from *Oryzias latipes* (Figure 6A). Yellow catfish *crt* clustered most closely with *Astyanax mexicanus* and *Danio rerio*, and more distantly from *Oryzias latipes* (Figure 6B). Based on the phylogentic analysis, all teleost ER stress–sensing genes (*perk*, *ire*-1α, and *atf*-6α) formed an independent cluster, whereas amphibian and mammalian corresponding proteins formed another cluster, clustering the yellow catfish most closely with *Astyanax mexicanus* and *Danio rerio*, and more distantly from *Oryzias latipes* (Figure 6, C–E).

### mRNA tissue expression of *grp78*, *crt*, *perk*, *ire*-1α, and *atf*-6α

**Figure 7:**
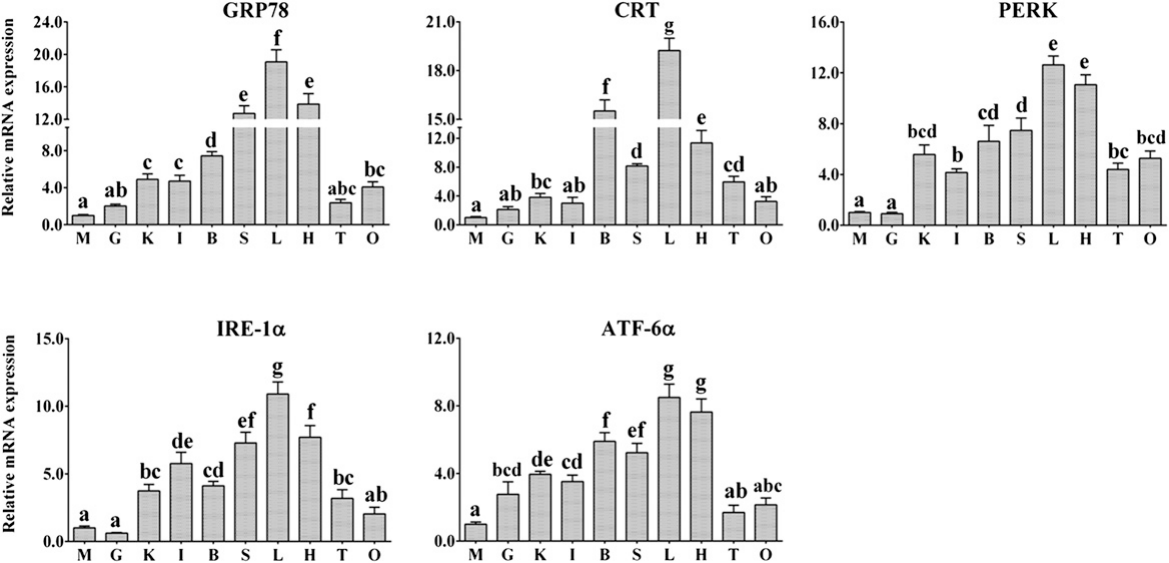
qPCR analysis for *grp78*, *crt*, *perk*, *ire*-1α, and *atf*-6α across skeletal muscle (M), gill (G), head kidney (K), mid-intestine (I), brain (B), spleen (S), liver (L), heart (H), testis (T), and ovary (O). Data (mean± SEM, n = 6) were normalized to housekeeping gene (β-*actin* and *gapdh*) expressed as a ratio of the control (value at skeletal muscle = 1). Bars that do not share a common letter are significantly different among the different tissues (*P* < 0.05).

The *grp78*, *crt*, *perk*, *ire*-1α, and *atf*-6α genes from *P. fulvidraco* were detected in all sampled tissues, but their mRNA abundance varied (Figure 7). *grp78* mRNA levels were the highest in liver, followed by heart, spleen, brain, head kidney, mid-intestine, and ovary, and were lowest in testis, gill, and skeletal muscle. *crt* mRNA levels were the highest in liver, followed by brain, heart, spleen, testis, and head kidney, and were the lowest in ovary, mid-intestine, gill, and skeletal muscle. *perk* mRNA levels were the highest in liver and heart, followed by spleen, brain, head kidney, ovary, testis, and mid-intestine, and were the lowest in skeletal muscle and gill. *ire*-1α mRNA expression is the highest in the liver, followed by heart, spleen, mid-intestine, brain, head kidney, and testis, and were the lowest in skeletal muscle and gill. *atf*-6α mRNA levels were the highest in liver and heart, followed by brain, spleen, head kidney, mid-intestine, and gill, and were the lowest in ovary, testis, and skeletal muscle.

### Effect of dietary Cu deficiency and excess on expression of *grp78*, *crt*, *perk*, *eif*2α, *ire*-1α, *xbp-1*, and *atf*-6α

**Figure 8:**
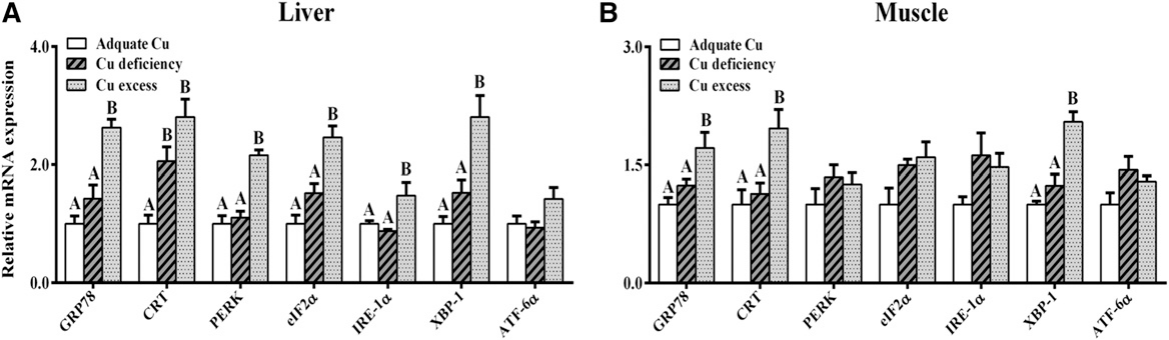
Effects of dietary Cu levels on the expression of *grp78*, *crt*, *perk*, *ire*-1α, and *atf*-6α mRNAs in the liver and muscle of yellow catfish after 8 wk were quantified by qPCR. Data (mean ± SEM, n = 3 replicate tanks, three fish were sampled for each tank) were normalized to housekeeping gene (β-*actin* and *gapdh*) expressed as a ratio of the control (value at adequate Cu = 1). Different letters indicate significant differences among the treatments (*P* < 0.05).

The effect of dietary Cu deficiency and excess on expression of *grp78*, *crt*, *perk*, *eif*2α, *ire*-1α, *xbp-1*, and *atf*-6α mRNAs in the liver and muscle of *P. fulvidraco* was shown in Figure 8. In liver, compared to dietary adequate Cu group, dietary Cu excess significantly upregulated mRNA expression levels of *grp78*, *crt*, *perk*, *eif*2α, *ire*-1α, and *xbp-1*, and dietary Cu deficiency only significantly upregulated mRNA expression levels of *crt* but showed no significant effects on other tested genes’ expressions. In muscle, compared to the dietary adequate Cu group, dietary Cu excess significantly upregulated mRNA levels of *grp78*, *crt*, and *xbp-1*, but showed no significant effects on mRNA expression of *perk*, *eif*2α, *ire*-1α, *xbp-1*, and *atf*-6α. mRNA levels of all seven tested genes showed no significant differences between dietary Cu deficiency group and dietary adequate Cu groups in skeletal muscle.

## Discussion

To date, the five full-length *grp78*, *crt*, *perk*, *ire*-1α, and *atf*-6α cDNAs have only been cloned in limited fish species, such as grass carp *Ctenopharyngodon idella* (*grp78*) (Zhu *et al.* 2013), rainbow trout *Oncorhynchus mykiss* (*crt*) (Kales *et al.* 2004), Asian seabass *Lates calcarifer* (*crt*) (Bai *et al.* 2012), and medaka *Oryzias latipes* (*atf*6) (Ishikawa *et al.* 2011). In this study, for the first time, we identified the full-length cDNA sequence of all five genes and explored their tissue distribution profiles from yellow catfish, and also indicated that the majority of their mRNA expression levels were differentially regulated by dietary Cu deficiency and excess, which would contribute to our understanding of the molecular basis of ER stress and would also greatly extend our understanding of the nutrition of Cu in fish.

In the present study, the ORF amino acid sequences of yellow catfish *grp78*, *crt*, *perk*, *ire*-1α, and *atf*-6α shared the high identity in the range of 91.9–94.9%, 69.9–84.8%, 56.8–74.4%, 65.1–81.7%, and 40.2–65.0% to those of other species, in agreement with other reports (Harding *et al.* 1999; Higashino *et al.* 2006; Ishikawa *et al.* 2011; Lenartowski *et al.* 2014; Das *et al.* 2015). The predicted amino acid sequences for the yellow catfish ER molecular chaperones (*grp78* and *crt*) revealed that the peptide contained all of the structural features that are characteristic of ER molecular chaperones in other species, containing 16- and 18-residue signal peptide sequences and the KDEL motif (Kales *et al.* 2004; Higashino *et al.* 2006; Bai *et al.* 2012; Das *et al.* 2015). The signal peptide at the N terminus and the consensus tetrapeptide KDEL at the C terminus of the amino acid sequence ensure the protein targeting to the ER. KDEL is a retrieval motif essential for the precise sorting of ER resident proteins along the secretory pathway. It is also known that *grp78* has two major domains, the N-terminal domain containing the ATPase catalytic site and the C-terminal substrate-binding domain (Gaut and Hendershot 1993; Gething 1999; Das *et al.* 2015), which are also conserved in the *grp78* of yellow catfish. Structural predictions of *crt* indicated that the protein had at least three domains, *i.e.*, N, P, and C domains. The P-domain of *crt* comprises a proline-rich sequence with three repeats of the amino acid sequence PXXIXDPDAXKPEDWDE followed by three repeats of the sequence GXWXPPXIXNPXYX. Similar results were also obtained in Japanese monkey (*Macaca fuscata*) (Higashino *et al.* 2006). The P-domain binds Ca^2+^ with high affinity, and the repeats may be essential for the high affinity of Ca^2+^ binding of *crt* (Fliegel *et al.* 1989; Ellgaard *et al.* 2001). The predicted amino acid sequence of the yellow catfish *perk* revealed that the peptide contained the structural features that are characteristic of *perk*, such as a signal peptide, a transmembrane domain, a cytosolic Ser/Thr kinase domain, sensor domain, and effector domain (Cui *et al.* 2011). *ire*-1α from mammals are characterized by an amino-terminal ER lumenal domain, a transmembrane region, and a carboxy-terminal domain that contains both kinase and RNase catalytic activities (Ishikawa *et al.* 2011), which are also conserved in the *ire*-1α of yellow catfish. The predicted amino acid sequence of *atf*-6α from yellow catfish and other species are characterized by a cytoplasmic N-terminus that contains a bZIP motif, which functions as a transcription factor following regulated intramembrane proteolysis in ER-stressed cells (Ishikawa *et al.* 2011). Phylogenetic analysis suggested that yellow catfish *grp78*, *crt*, *perk*, *ire*-1α, and *atf*-6α peptides were closely related to other fish with similar taxonomic classes, similar to those reported in other fish (Kales *et al.* 2004).

The analysis of tissue distribution profiles for genes is useful to better understand their physiological roles. At present, in fish several studies have been involved in mRNA expression of *grp78* (Hori *et al.* 2010; Zhu *et al.* 2013; Rebl *et al.* 2014; Das *et al.* 2015) and *crt* (Kales *et al.* 2004; Bai *et al.* 2012), and other reports in mRNA expression profiles of ER stress-relevant transcripts in fish (Chen *et al.* 2014; Komoike and Matsuoka 2013). However, the mRNA expression profiles of *perk*, *ire*-1α, and *atf*-6α were still absent in fish. The present study indicated that *grp78* mRNA levels were the highest in liver, followed by heart and brain. The noninduced level of expression of *grp78* indicated that it might play multiple functions in yellow catfish as its homologs reported in other living organisms (Xi and Ma 2013). Zhu *et al.* (2013) indicated that the expression of *grp78* in the liver of grass carp *Ctenopharyngodon idella* was higher than other tissues. Other studies also indicated higher *Grp78* accumulation in brain (Higashino *et al.* 2006; Xi and Ma 2013) and heart tissue in mammals (Oh-hashi *et al.* 2003). In the present study, *crt* mRNA was expressed ubiquitously in every tissue, with the highest in liver and the lowest in ovary, mid-intestine, gill, and skeletal muscle. Kales *et al.* (2004) reported the ubiquitous expression of *crt* mRNA in all tissues tested, with highest expression in liver. In Asian seabass, Bai *et al.* (2012) observed that the highest expression was seen in the liver, followed by that in the brain, spleen, and kidney. In Japanese monkey, Higashino *et al.* (2006) pointed out that *Crt* mRNA was expressed ubiquitously in every tissue except the thymus and skeletal muscle, which had low expression. The present study also detected the low *crt* mRNA expression in the intestine, in agreement with the reports by Kales *et al.* (2004). The present study indicated the *Crt* mRNA levels in testis. *crt* has been identified in the sperm of several mammals (Ho and Suarez 2003; Naaby-Hansen *et al.* 2001) and appears to be involved in regulating sperm capacitation, motility, and acrosome reaction (Naaby-Hansen *et al.* 2001; Ho and Suarez 2003). Like mammals (Koyabu *et al.* 1994) and rainbow trout (Kales *et al.* 2007), yellow catfish *crt* may play only a minor role in skeletal muscle, conserving its role in calcium modulation for smooth muscle such as the heart (Waisman *et al.* 1986). The present study reported the ubiquitous mRNA expression of the three ER stress sensors (*perk*, *ire*-1α, and *atf*-6α) in all tested tissues, with the highest expression in liver and the lowest in skeletal muscle and gill. At present, mRNA expression profiles of these genes in fish were very scarce. Studies pointed out that the expression level of ER stress proteins was expected to be high in secretory cells, as these cells are rich in ER (Palade 1975). Their commonly high expression in the liver, spleen, and head kidney is consistent with this hypothesis. The present study also indicated the high expression of ER stress–related genes was found in the heart. Studies suggested that ER stress might protect the heart and even foster hypertrophic growth of the myocardium in mammals (Okada *et al.* 2004; Mao *et al.* 2006).

In mammals, lipid droplet accumulation is often induced by ER stress (Ozcan *et al.* 2004; Oyadomari *et al.* 2008). Studies indicated Cu could act as a modifier in lipid metabolism (Burkhead and Lutsenko 2013) and the variations in dietary Cu have a profound effect on lipid deposition in fish (Berntssen *et al.* 1999; Tan *et al.* 2011; Shaw and Handy 2006). Recently, we have indicated that dietary deficiency and excess could influence the key lipid metabolism–related genes expression (*i.e.*, *6pgd*, *g6pd*, *fas*, *accα*, *pparγ*, *lxr*, *hsl*, *pparα*, and *atgl*) and enzyme activities (*i.e.*, 6pgd, G6pd, and Fas) in yellow catfish (Chen *et al.* 2015). To shed light on the molecular mechanisms underlying the change of lipid storage in yellow catfish, we analyzed the changes of mRNA expression of these ER-related genes known to play an important molecular role in ER stress–induced changes of lipid deposition. The present study, for the first time, found dietary Cu excess activated ER stress and the UPR signaling in a tissue-specific manner in yellow catfish. First, the chaperones and folding sensors in ER, including *grp78* and *crt*, were significantly upregulated in liver and skeletal muscle of *P. fulvidraco* in dietary Cu excess groups. Upregulation of *grp78* and *crt* often acts as an index of ER stress (Lee 2005). Studies reported marked upregulation of ER chaperones such as *grp78* and *crt* in response to metal ions (Liu *et al.* 2006; Shinkai *et al.* 2010; Zhu *et al.* 2013). Second, we found that two UPR branches, the *perk*–*eif*2α pathway and the *ire*1–*xbp1* pathway, were activated by dietary Cu excess in liver of *P. fulvidraco*. Studies also suggested that under ER stress, UPR pathways, *i.e.*, *perk*–*eif*2α and the *ire*1–*xbp1*, are activated to reduce the ER load of the unfolded proteins (Ron and Walter 2007). In LLC-PK1 cells, Liu *et al.* (2006) found *eif*2α mRNA levels were elevated in response to CdCl_2_ exposure. The activation of UPR pathways will in turn influence lipid metabolism because these pathways control hepatic lipogenesis, as suggested by several studies (Lee *et al.* 2008; Oyadomari *et al.* 2008; Lee and Glimcher 2009). *atf*6 also plays an important role in regulating hepatic lipid homeostasis (Yamamoto *et al.* 2007; Rutkowski *et al.* 2008). In the present study, dietary Cu excess did not significantly influence *atf*6α expression, which showed that the *atf*6α pathway was not the main branch for the Cu-induced ER stress. Considering the lack of change in ER stress–related genes’ expressions in skeletal muscle following dietary Cu treatments, we believe this lack of response in these genes’ expression levels suggests a different mode of regulation or function in fish skeletal muscle. Because the ER stress plays important roles in lipid homeostasis, the ER stress and UPR may be the fundamental reaction to the dietary Cu exposure. Thus, ER stress may be the unrecognized mechanism underlying the changes of lipid deposition that are common to dietary Cu levels.

In summary, we cloned the full-length sequences of five key genes (*grp78*, *crt*, *perk*, *ire*-1α, and *atf*-6α) involved in ER stress response from yellow catfish and investigated their molecular characterization, which will facilitate further studies on the regulation of ER stress response at the molecular level. mRNA expression patterns of these genes in different tissues also provided a basis for further exploring the differential regulatory patterns of ER stress response in different tissues for the fish species. Dietary Cu excess activated ER stress and the UPR signaling in a tissue-specific manner in yellow catfish, which will help us to understand the effects of dietary Cu levels on the upstream pathway of lipid metabolism (the ER) and thus provide some novel insights on the nutrition of Cu in fish.
